# Drug-Loaded Polymeric Particulated Systems for Ophthalmic Drugs Release

**DOI:** 10.3390/molecules27144512

**Published:** 2022-07-14

**Authors:** Ruxandra Mihailovici, Alexandra Croitoriu, Florin Nedeff, Valentin Nedeff, Lacramioara Ochiuz, Decebal Vasincu, Ovidiu Popa, Maricel Agop, Andreea Moraru, Danut Costin, Marcel Costuleanu, Liliana Verestiuc

**Affiliations:** 1 Faculty of Medicine, Grigore T. Popa University of Medicine and Pharmacy, 16 Universitatii Street, 700115 Iasi, Romania; ruxandra.mihailovici@gmail.com (R.M.); croitoru.alexandra@icmpp.ro (A.C.); costin_danut@yahoo.com (D.C.); marcel.costuleanu@umfiasi.ro (M.C.); 2 Faculty of Medical Bioengineering, Grigore T. Popa University of Medicine and Pharmacy, 16 Universitatii Street, 700115 Iasi, Romania; liliana.verestiuc@bioinginerie.ro; 3 Department of Industrial Systems Engineering and Management, Faculty of Engineering, “Vasile Alecsandri” University of Bacau, 600115 Bacau, Romania; 4 Department of Environmental Engineering and Mechanical Engineering, Faculty of Engineering, “Vasile Alecsandri” University of Bacau, 600115 Bacau, Romania; vnedeff@ub.ro; 5 Department of Pharmaceutical and Biotechnological Drug Industry, Grigore T. Popa University of Medicine and Pharmacy, 700115 Iasi, Romania; lacramioara.ochiuz@umfiasi.ro; 6 Department of Natural, Bioactive and Biocompatible Polymers, Petru Poni Institute of Macromolecular Chemistry, Aleea Grigore Ghica Voda 41A, 700487 Iasi, Romania; decebal.vasincu@umfiasi.ro; 7 Department of Emergency Medicine, Faculty of Medicine, Grigore T. Popa University of Medicine and Pharmacy, 700115 Iasi, Romania; ovidiu.popa@umfiasi.ro; 8 Department of Physics, “Gh. Asachi” Technical University of Iasi, 700050 Iasi, Romania; 9 Romanian Scientists Academy, 050094 Bucharest, Romania

**Keywords:** particle-based drug delivery, pre-emulsions, routes of administration, ophthalmic drugs, fractal mathematical model

## Abstract

Drug delivery to the anterior or posterior segments of the eye is a major challenge due to the protection barriers and removal mechanisms associated with the unique anatomical and physiological nature of the ocular system. The paper presents the preparation and characterization of drug-loaded polymeric particulated systems based on pre-emulsion coated with biodegradable polymers. Low molecular weight biopolymers (chitosan, sodium hyaluronate and heparin sodium) were selected due to their ability to attach polymer chains to the surface of the growing system. The particulated systems with dimensions of 190–270 nm and a zeta potential varying from −37 mV to +24 mV depending on the biopolymer charges have been obtained. Current studies show that particles release drugs (dexamethasone/pilocarpine/bevacizumab) in a safe and effective manner, maintaining therapeutic concentration for a longer period of time. An extensive modeling study was performed in order to evaluate the drug release profile from the prepared systems. In a multifractal paradigm of motion, nonlinear behaviors of a drug delivery system are analyzed in the fractal theory of motion, in order to correlate the drug structure with polymer. Then, the functionality of a SL(2R) type “hidden symmetry” implies, through a Riccati type gauge, different “synchronization modes” (period doubling, damped oscillations, quasi-periodicity and intermittency) during the drug release process. Among these, a special mode of Kink type, better reflects the empirical data. The fractal study indicated more complex interactions between the angiogenesis inhibitor Bevacizumab and polymeric structure.

## 1. Introduction

In the field of ophthalmology, the delivery of the drugs to the target region may be considered a real challenge because of the eye anatomy and physiological barriers that serve as protective mediums of the eye and the non-compatibility of such barriers with molecular volume of the most pharmacological agents [1].

Topical application of the drugs is the preferred route, even the target tissues are in the back part of the eye. The intravitreal route is also used to deliver therapeutic entities to posterior tissues, but this route can lead to retinal detachment or endophthalmitis. Iontophoresis has been shown to increase the transscleral permeability of many drugs, including fluorescein, antibiotics and antivirals [2]. However, the technique is unable to deliver significant amounts of macromolecular drugs to the vitreous or retina [3]. Direct administration using any of these routes faces many problems related to drug bioavailability, including side effects and potential toxicity because of the repeated treatments to achieve therapeutic drug levels [4]. In this regard, the challenge of using particulate systems includes improved topical passage of large, poorly water-soluble drugs for immune-related and vision-threatening diseases or unstable molecules, such as nucleic acids, for gene transfer therapy in the retinal diseases [5,6,7,8]. Recently, new carrier systems such as micelles, liposomes, micro and nanoparticles (NPs) and dendrimers have been under investigation for delivery of therapeutic agents to the eye [9,10,11]. Moreover, fully synthetic materials such as poly(ε-caprolactone), poly(cyanoacrylate)s or poly(ethylene glycol), chitosan and poly(acrylic acid) have been recently investigated as coatings polymers with potential applications at the ocular level because of their biocompatibility, electrical charges which assure adhesion properties and pH responsivity [12,13,14].

Chitosan is a cationic, biocompatible and biodegradable polymer of natural origin (obtained by deacetylation of chitin extracted mainly from crab shells) that has good release characteristics and biological properties that make it a promising candidate for use in ocular drug delivery [15,16,17]. The positive effect of the materials based on chitosan has been attributed to an improved adherence to the ocular mucosa and entrance to the superficial layers of the cornea and conjunctiva [18,19]. The formulation of biodegradable polymers as colloidal or particulate systems is a promising research direction in ophthalmic drug delivery. Generally, it is accepted that a colloidal system can be loaded with low soluble drugs, and this permits drop-wise administration while preserving the drug activity for the biological site [20]. However, no procedure regarding the formulation of drug-loaded particulate systems has been standardized, although several there are reported as synthetic methods and drug loading techniques. The major developmental issues in the case of particulated systems include particle size uniformity, formulation stability, drug release control rate and manufacturing sterile preparations for large scale systems [21,22]. To increase the efficiency of uptake, certain modifications have been investigated, including conjugation with specific ligands such as thiamine [23] or transferrine [24], or coating with surfactant [25].

The drug delivery technology offers a high level of safety over continuous release time and for an extended period of time, in controllable amounts, with maintaining the bioactivity of the drug [26]. In ophthalmic applications, the drug release from particulated formulation depends on some parameters such as pH, temperature, drug solubility, drug diffusion through particles matrix and biodegradation of polymers [27]. The specific properties of polymeric particles, such as biocompatibility, biodegradability and reduced size, the correlation of the physical–chemical properties of the drugs and those of the chemical substances used in the synthesis of the particles are considered in preparing drug delivery systems with predictable functionality in ophthalmic environment. The formulation properties undergo changes depending on their preparation methods used, thus leading to a multitude of applications in the biomedical field [28,29,30].

The paper presents the preparation and characterization of drug-loaded polymeric systems for few ophthalmic drugs, considering polymers with various electrical charges and layer-by-layer method to formulate particulated materials, and analyzes the release profile of the drug. In order to select the optimal composition, stable over time pre-emulsion incorporating drugs with specific therapeutic action at the eyeball level was prepared and coated with biodegradable polymers. All selected biopolymers (chitosan, sodium hyaluronate and heparin sodium) have low molecular weight in order to facilitate polymer chains attachment to the surface of growing system. An extensive modelling study was performed in order to evaluate the drug release profiles. In a multifractal paradigm of motion, nonlinear behaviors of a drug delivery system were analyzed in the fractal theory of motion. Then, the functionality of a SL(2R) type “hidden symmetry” implies, through a Riccati type gauge, different “synchronization modes” during the drug release process. Among these, a special mode of Kink type, better reflects the empirical data.

## 2. Results

### 2.1. Particulate Systems Preparation Using Layer-by-layer Technique

**Figure 1:**
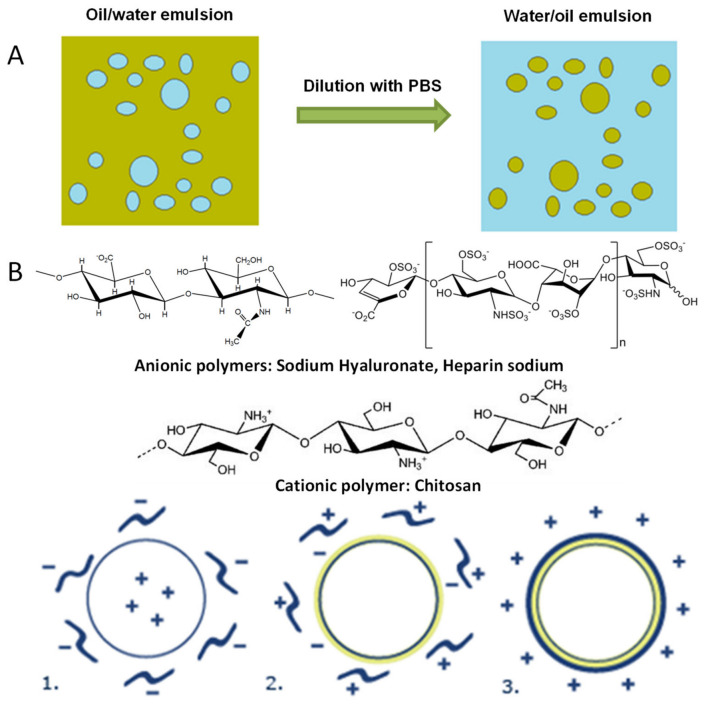
Coating of particles with the layer-by-layer technique: (**A**) pre-emulsion preparation and (**B**) pre-emulsion coating with polymers. 1. Sodium hyaluronate/heparin sodium attachment to the surface of the nucleus of vitamin A/E; 2. attaching chitosan to the particles surface; 3. final particle.

Particulate systems with a hydrophobic core (vitamin E/vitamin A) and coated with biopolymers (sodium hyaluronate/sodium heparine and chitosan) and drugs (pilocarpine, dexamethasone phosphate and bevacizumab) have been prepared by a pre-emulsion formation (phase inversion point) and coating with the biodegradable polymers by the layer-by-layer method (Figure 1).

By the phase reversal point method, a Gaussian distribution of the initial pre-emulsion was obtained, with an average diameter of 209 nm and a PDI (polydispersity index) of 0.281 for the systems containing vitamin A/vitamin E as active ingredient.

The passage through the lamellar phase led to a strong increase of the viscosity in the phase reversal process. This transition was also characterized by a significant increase in system temperature by up to + 10 °C. After completion of the infusion, the resulting system presented a milky-white appearance and a relatively low viscosity. The high density of particles in the emulsion determined the subsequent dilution, preceding the addition of the polymer solutions, in order to facilitate a more efficient deposition of the macromolecules by increasing the distance between internal particles. According to the data in the literature [31], stable emulsions assume particle sizes below the limit of 500 nm and PDI below 0.3. The potential zeta obtained was + 8.37 mV, indicating the cationic character (cationic emulsion) of the system and the possibility of the subsequent addition of polymers to the surface.

### 2.2. The Influence of CTAB Concentration on the Emulsion Characteristics

The solitary use of the nonionic surfactant imparts a negative zeta potential to the particles for the following reason: the polyoxyethylene residues of Tween 80 are strongly hydrated by the aqueous phase of the emulsion. This hydration is due to the van der Walls bonds as well as the multiple hydrogen bonds formed by these residues with water molecules. It has been found that OH^−^ and H_3_O^+^ ions have a different affinity for polyoxyethylene residues, in the sense that more hydrogen bonds are established with hydroxyl ions than with hydronium ions. As a result, the surface charge of the globules of an oil-in-oil emulsion, stabilized with Tween 80 will be negative, a phenomenon confirmed by the zeta potential values. The potential zeta for the emulsion based on Tween 80 (PE0) was −6.92 mV (Table 1).

Cetyl trimethyl ammonium bromide is a cationic surfactant, and its presence in emulsion leads to particles with a positive surface charge. As the proportion of CTAB in the emulsifier mixture increases, the positive surface electric charge of the dispersed phase particles increases too. According to the data obtained, minimum sizes were recorded for a percentage of 2% CTAB. However, this has led to an increase in the polydispersity index, but it remained under the accepted value of 0.3. This index is relevant for characterizing the Gaussian distribution of dimension values. There is a considerable increase in zeta potential values with increasing CTAB concentration, but higher concentrations of surfactant are not indicated due to the harmful effect on the cell membrane [32]. The pre-emulsion PE2 was selected for further experiments.

**Figure 2:**
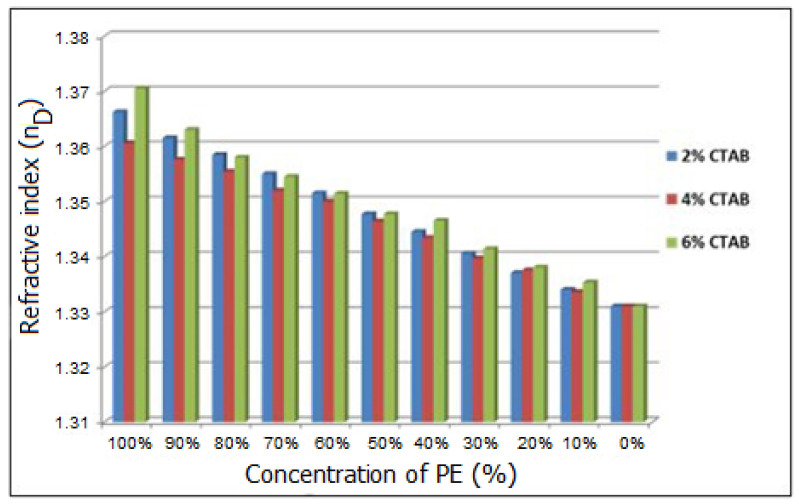
Refractive index for diluted pre-emulsions prepared with different proportions of CTAB.

The measurement of the refractive index of the systems can be associated with possible discomfort or visual impairments for patients after the eye drops instillation. The refractive index of the tear fluid varies between 1.340 and 1.360 [33] and correlated with these values it is recommended as eye drops to not have refractive index values higher than 1.476 [34]. In the case of the samples analyzed, even for undiluted samples, the index is approaching to that of the tear fluid. It is also noted that no concentration exceeds the maximum allowed value (Figure 2).

### 2.3. Characterization of Synthesized Particulated Systems

**Figure 3:**
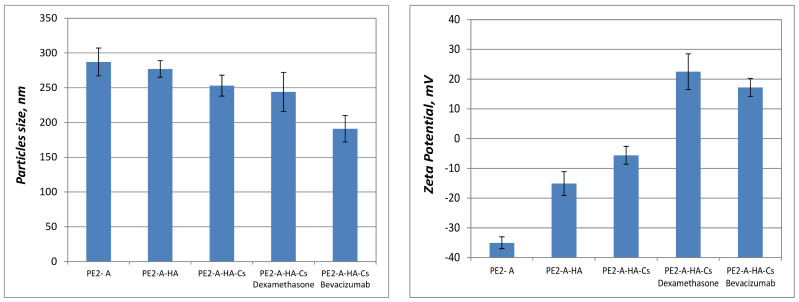
Size (nm) and zeta potential (mV) for pre-emulsion prepared with vitamin A (PE2-A), particles coated with sodium hialuronate (PE2-A-HA), particles coated with sodium hialuronate and chitosan (PE2-A-HA-Cs) and particles coated with biopolymers and dexamethasone/bevacizumab (PE2-A-HA-Cs-Dexamethasone/Bevacizumab).

**Figure 4:**
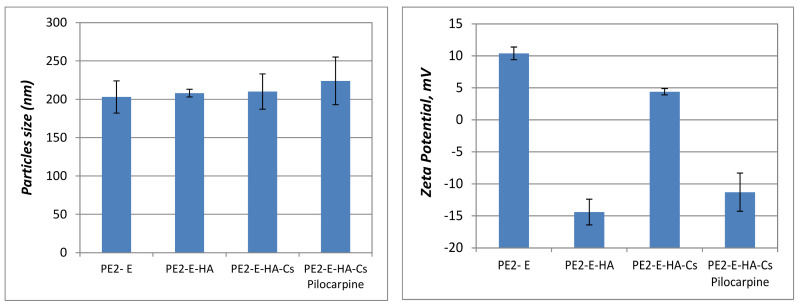
Size (nm) and zeta potential (mV) for pre-emulsion prepared with vitamin E (PE2-E), particles coated with sodium hialuronate (PE2-E-HA), particles coated with sodium hialuronate and chitosan (PE2-E-HA-Cs) and particles coated with biopolymers and pilocarpine (PE2-E-HA-Cs-Pilocarpine).

The particles size and zeta potential after coating with biodegradable polymers (sodium hyaluronate/heparin sodium). after the addition of polymer layers and loaded with drugs are synthesized in Figure 3 and Figure 4.

In the case of the particles based on vitamin A and hyaluronic acid, the dimensions increase after each step of adding polymers, but in the case of the particles with vitamin E and heparin content, there are insignificant differences in terms of size distribution. For the values of the zeta potential, the particles containing vitamin A, hyaluronic acid, chitosan and dexamethasone, an increase in positive surface can be observed, with the addition of the positive charged chitosan layer. The particles containing vitamin E, sodium hialuronate, chitosan and pilocarpine, both an increase and a decrease of the surface charge are identified after each layer was added.

Regarding the time stability of the particulate systems, a very small variation of the particle size of the internal phase of cationic emulsion was observed. It varied upwards by a maximum of 30 nm over a period of 8–12 months, the samples being stored at room temperature in parafilm sealed containers. In the case of the particles, a slight destabilization was observed within a few days for keeping at room temperature. High dilution of the pre-emulsion and the polymers addition, especially in the case of chitosan and sodium hyaluronate, have promoted the rapid proliferation (several days) of invasive microorganism cultures. By contrast, the preservation of the samples that were prepared with heparin and stored in a refrigerator (6–8 °C) showed high resistance to microorganisms and insignificant changes in the size and zeta potential for periods of up to 3–4 months.

### 2.4. FT-IR Data

**Figure 5:**
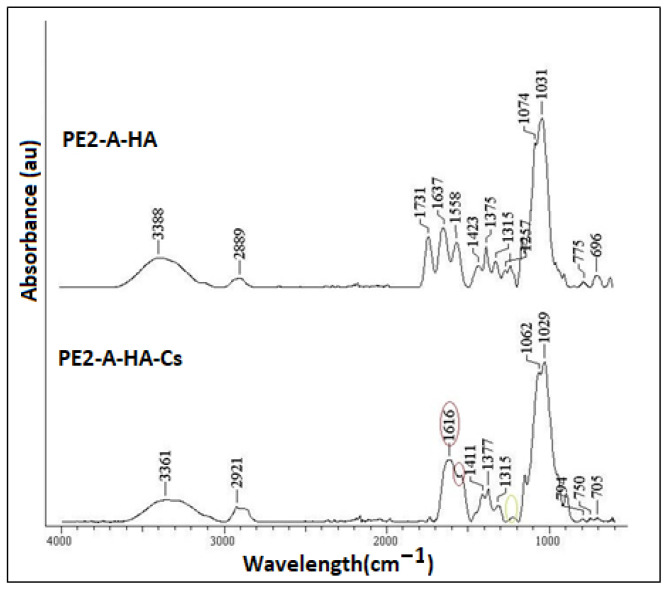
FTIR data for PE2-A-HA and PE2-E-HA-Cs particles.

The following absorption bands are recorded for HA (Figure 5): the band at 3388 cm^−1^ corresponds to the stretching vibration of the alcoholic OH group and to the stretching vibration of the hydrogen bond corresponding to the N-H group, respectively; the band from 2889 cm^−1^ corresponds to the asymmetric stretching vibration corresponding to the CH_2_ group; the 1731 cm^−1^ band corresponds to the stretching vibration of the free COOH group; the vibrations from 1637 cm^−1^ and 1423 cm^−1^ are asymmetric and symmetrical vibrations of the COO- group, respectively; the vibration at 1558 cm^−1^ corresponds to the NH group, and the vibration at 1031 cm^−1^ corresponds to the C-O-C saccharide units in the hemiacetal system. CS shows increased the bands from 1616 cm^−1^ and 1591 cm^−1^ representative for the primary amide, respectively, the secondary amide (C=O), and the band from 1411 cm^−1^ corresponds to the symmetrical deformation of the group (C-H). At 1377 cm^−1^ is the band characteristic of the stretching vibration of the C–N bond, while the band at 1060 cm^−1^ is attributed to the absorption vibration of the group (C-O-C). All these data confirmed the polymer coatings.

### 2.5. In Vitro Drug Release

**Figure 6:**
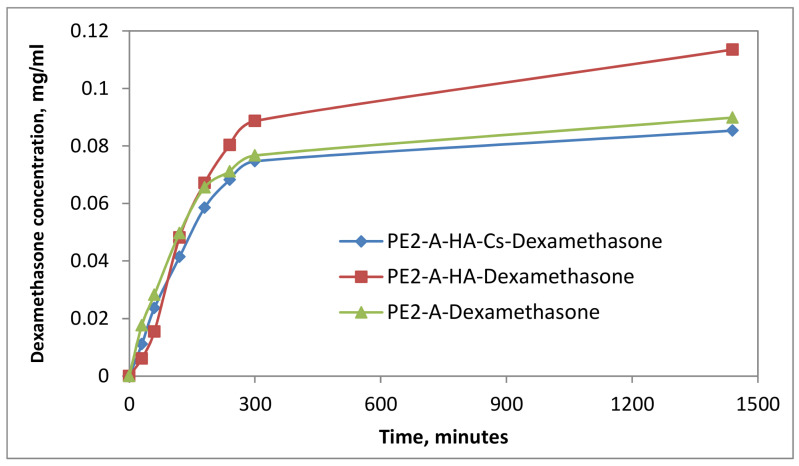
Kinetic curves of dexamethasone release from particulate systems.

**Figure 7:**
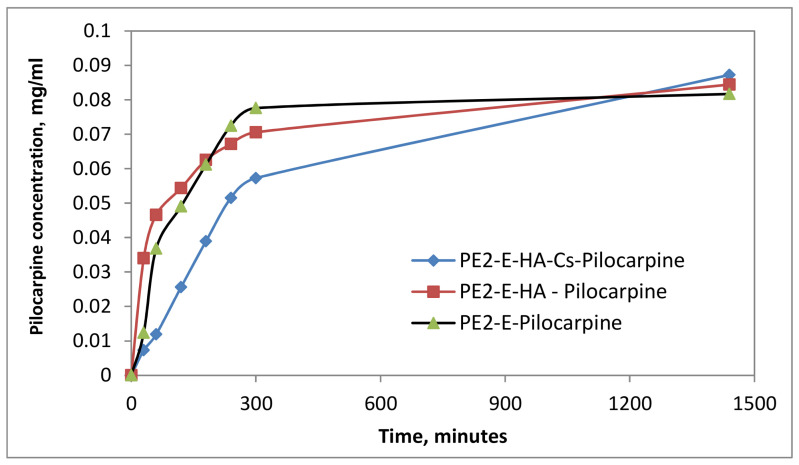
Kinetic curves of dexamethasone release from particulate systems.

**Figure 8:**
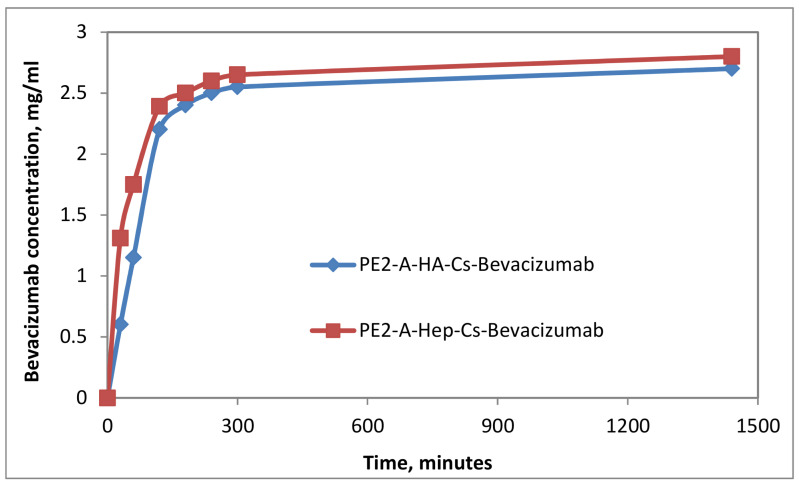
Kinetic curves of Bevacizumab release from particulate systems.

**Figure 9:**
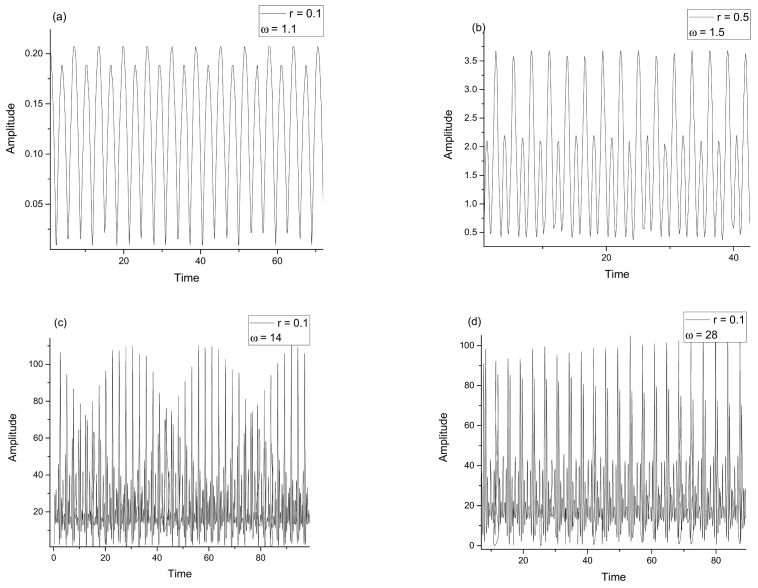
Dependences with time of Re z for different values of the ω and r: (**a**) ω=1.1, r=0.1; (**b**) ω=1.5, r=0.5; (**c**) ω=14, r=0.1; (**d**) ω=28, r=0.1. Release dynamics through multifractal self-modulation at a non-differentiable scale in the form of period doubling, quasi-periodicity, damped oscillations and intermittency.

The drug release assay was performed under physiological conditions, specific to the extracellular environment: 37 °C and pH = 7.4. From the data it can be noted that PE2-A/E emulsions released the drug in the firsts 200–360 min (4–6 h), and after that a plateau was registered (Figure 6, Figure 7 and Figure 8). The particulate systems with HA exhibited a slower release. By comparison the systems with HA and Cs led to a delayed profile, similar to a retard effect, a prolonged release, which is more indicated in ophthalmic drug delivery. In Figure 9a,b some examples of dynamic releases predicted by the multifractal model over a controllable period of time are represented. The model captures a dynamics which is defined by a period doubling evolution. It means that in the multifractal plane the release of a high concentration of drug is succeeded by a lower dose. In Figure 9c,d, where a different interaction scale was chosen, we see alternatives for drug release dynamics, alternatives which embody a more complicate and complex dynamics [35]. Certain conditions reveal a damped oscillatory scenario which can be used in understanding the long-time evolution of drug release, while on a select window of fractal parameters release through intermittence with a controllable modulating frequency can be achieved. Finally, the last release scenario that can be simulated by the multifractal model sees a modulated dynamic which can be controlled in terms of concentration and frequency. Within the vast complexity of a multifractal model for drug release we were able to capture small space-time scale dynamics of the drug release with controllable dynamics, which can be further used to control and improve the overall drug release dynamics.

**Figure 10:**
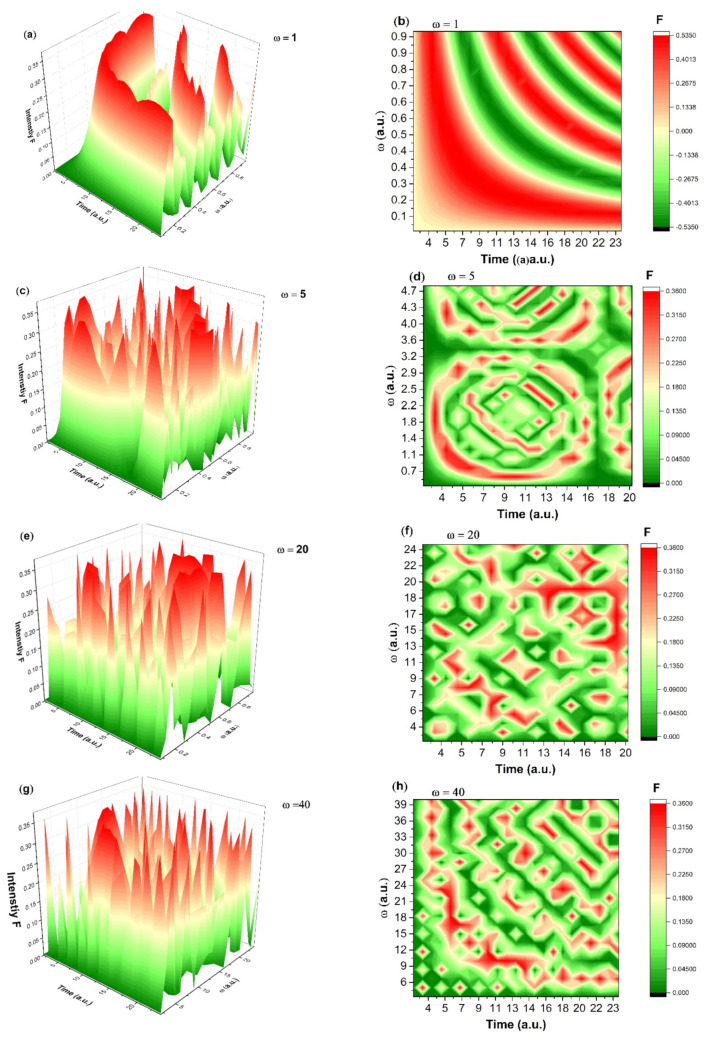
3D and 2D dependences of Re z for different values of ω and t at a constant value of (**a**,**b**) ω=1, r=0.3; (**c**,**d**) ω=5, r=0.3; (**e**,**f**) ω=20, r=0.3; (**g**,**h**) ω=40, r=0.3.

Both 3D and the corresponding 2D dependence of Re z on Ω and t parameters are represented in Figure 10. It can be seen that the evolution for each particular scale resolution involves a self-modulation which is strongly dependent on the selection of the scale. The damped drug release scenario is seen for small values of the scale resolution, while its increase leads to intermittent and modulated response of the system. At large scale resolutions the system is seen to have a complex release scenario which depends on the physical properties of the polymer matrix and the biological release conditions. This result is also consistent with empirical data which are taken at specific points, and thus disregard the small scale complex dynamics. As the model is of multifractal nature in specific conditions seen in Figure 10c,d quasi-chaotic releases can be generated as a route for drug release. This region is obviously unwanted in real systems. It is worth noted that the system, while it transitions towards chaos exhibiting some particular properties, it never reaches a chaotic state, but it jumps back to the damped oscillatory regime.

### 2.6. HET-CAM Tests

**Figure 11:**
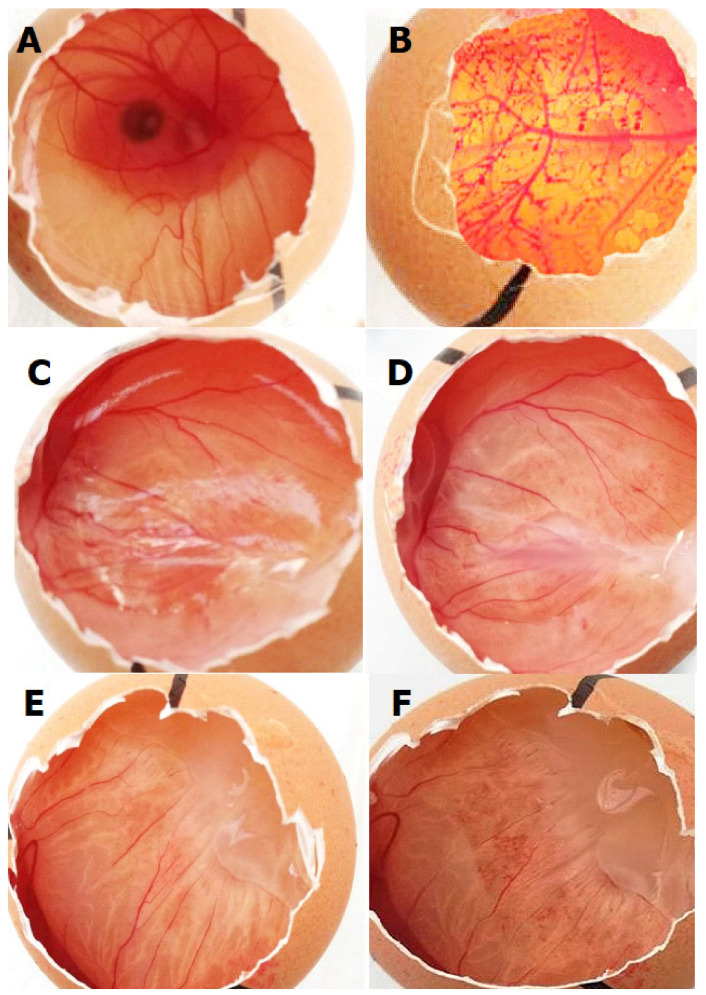
Effects of the particulate systems on the chorioallantoic membrane: (**A**) negative control; (**B**) positive control; chorioallantoic membrane before (**C**) and after (**D**) application of PE2-A-HA-Cs-Dexamethasone; chorioallantoic membrane before (**E**) and after (**F**) application of PE2-A-HA-Cs-Bevacizumab (2×).

The chicken egg chorioallantoic membrane test was performed by testing two particulate systems: PE2-A-HA-Cs-Dexamethasone and PE2-A-HA-Cs-Bevacizumab (Figure 11).

The analysis of the results was carried out by means of images, determining the severity of any adverse reaction on a scale from 0 (no reaction) to 21 (strong reaction). The analysis of the standard samples indicated a strongly irritating reaction, only after the addition of the 1% sodium hydroxide solution, compared to the visual result obtained after the addition of the 3% saline solution. The irritation potential of the test emulsions was calculated by determining the time of onset of lysis, hemorrhage and coagulation, resulting in an irritation score in the range 0–0.9. The analysis of the images shows that the PE2-A-HA-Cs-Dexamethasone and PE2-A-HA-Cs-Bevacizumab, respectively, after a period of 5 min after application, caused a slightly hyperemia on the blood vessels in the chorioallantoic membrane. Due to the extended reaction time (after 5 min), the irritation score obtained had values that included the tested emulsions in the “non-irritating” spectrum.

## 3. Discussions

Current therapeutic procedures based on drug-loaded particles consist of topical instillation, intracameral or subconjunctival injection, depending on the target site. Such administration methods release the drug controlled and gradual and are alternatives to formulations that are using large and repeated doses of the active substance. Depending on the carrier properties and the physical–chemical characteristics of the drugs, they can reach only external layers (cornea, conjunctiva or sclera), internal (aqueous humor, iris, ciliary body, vitreous humor or retina) or both after topical instillation or intraocular administration [36,37]. However, only 1–7% of the administered drugs can reach the aqueous humor level because of tear distribution and conjunctival and cornea barriers [38,39]. In the present study, polymeric particulate systems are studied in terms of efficiency as carriers of drugs with applicability in ophthalmology and other medical fields (dexamethasone/pilocarpine/bevacizumab).

Various studies indicated that the small dimensions particles are efficient in ophthalmic applications if they do not affect the visual processes, are absorbed and metabolized without producing toxic products. The double emulsion crosslinking technique provided stable particles in shape and size (50–500 nm), having a high drug diffusion capacity [8,10]. In this context the paper aims to make a contribution in the vast field of ophthalmology by analyzing the possibility of obtaining intraocular complex polymer–drug systems, which have the ability to deliver active substances that target the main pathological processes. The benefits of prolonged and targeted bioavailability and drug delivery capability for treating ophthalmic disorders are often associated with chitosan/hyaluronic acid presence in particles. Polymer/drug-based particles indicate a wide range of advantages in the treatment of diseases of the anterior ocular segment due to the reduced size, penetration capacity of ocular barriers, mucoadhesiveness and biocompatibility [40]. These polymeric systems have the ability to create a good contact with the corneal and conjunctival surfaces, improving the drugs release profile to the external ocular tissues and limit the toxicity of the drugs on the internal ocular tissues and blood flow (through systemic absorption). The mucoadesivity nature of the chitosan is closely linked to the positively charged amino groups and their interactions with negatively charged residues of sialic acid in corneal and conjunctival mucus, which significantly increases the ocular bioavailability [41]. Other features of chitosan are related to the barrier penetration function, the ability to transient open the tight junctions between cells, their degradation in lacrimal fluid and controlled release of a variety of ophthalmic drugs [42].

The polymeric-based of controlled release systems for the anterior ocular segment are designed to improve the bioavailability of drugs in the extraocular tissues to alleviate the symptoms caused by ocular inflammatory surface or cornea disorders, such as dry eye syndrome and allergic conditions; additionally, for an increase bioavailability of drugs from intraocular tissues to treat infections and complex diseases, which may endanger vision, such as glaucoma or intraocular inflammation, and even retina diseases [43]. In this regards some combinations of polymers were developed and the present study offers results that are in agreement with the existing data in the literature. Coated emulsions with natural and biocompatible polymer (hyaluronic acid and chitosan) have been formulated and tested. The rate of dexamethasone/ pilocarpine/bevacizumab release can be changed by varying the polymer characteristics and drug/polymer ratio.

According to the literature data, stable emulsions involve particle sizes below the limit of 500 nm and the PDI below the value of 0.3 [44,45]. The prepared particles in this study have dimensions under 300 nm and the zeta potential can be varied from negative to positive one, based on the anionic/cationic character of the core emulsion and the polymers added on the surface: hyaluronic acid-negative one and chitosan-positive one. The particles release drugs in a safe and effective manner, without toxic effects, thus maintaining therapeutic concentration for a longer period of time.

An extensive modelling study was performed in order to evaluate the drug release profile from the prepared polymeric systems. Using the multifractal paradigm of motion, different synchronization modes (period doubling, damped oscillations, quasi-periodicity and intermittency) during the drug release process are established. Among these, a special mode of Kink type, better reflects the empirical data. Multifractal Riccati’s type Equation (1) has the bounded solution:

(1) $$\frac{A}{{\left(AC+B^{2}\right)}^{\frac{1}{2}}}\tanh^{-1}\frac{M-B}{{\left(AC+B^{2}\right)}^{\frac{1}{2}}}=\tau$$

**Figure 12:**
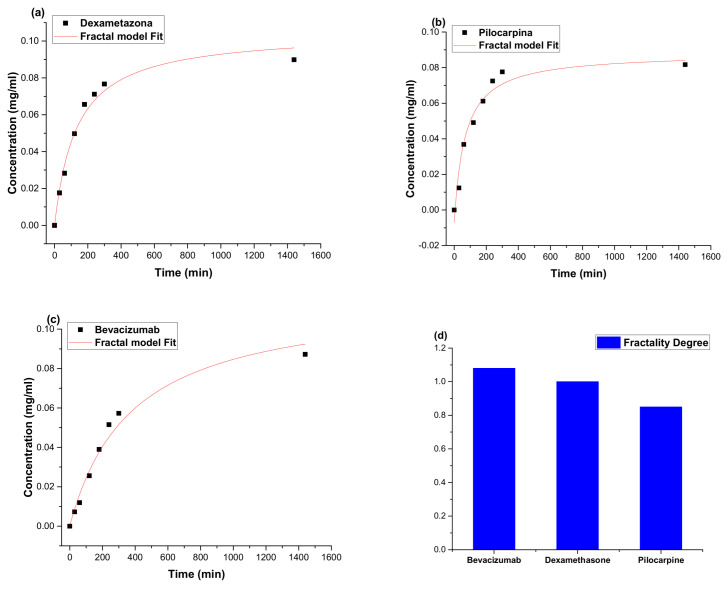
Theoretical fitting of the empirical data and an estimation of the fractality degree for each drug investigated (**a**–**c**) and the fractality degree evolution with the nature of the drug (**d**).

If we set $\equiv {\left(AC+B^{2}\right)}^{\frac{1}{2}}$, then

(2) $$M\left(t\right)=B+a\tanh\left(\frac{a\tau }{A}\right)$$

a relation that can describe the standard dynamics of release at various differentiable scale resolutions, through a convenient choice of variables and parameters (Figure 12).

The complexity of particle–drug system generates a complex release profile for drug. It is known that the complex trajectory can be measured through fractal dimension, namely, in this particular case, the fractal dimension of the release curve and is confirmed by fractal dimension values (fractality degree). The fractality degree decreased from the systems with bevacizumab to particles with dexamethasone and the lowest values were obtained for the particles with pilocarpine. The results indicate more complex interactions between the angiogenesis inhibitor Bevacizumab and polymeric structure.

## 4. Materials and Methods

### 4.1. Materials

The initially hydrophobic core included active liposoluble vitamins (vitamin Aretinol acetate, 1,500,000 UI/g, *Biofarm S.A* (Bucharest, Romania); vitamin E-α-Tocopherol acetate, 1360 IU/g from *Sigma Aldrich*^®^ (St. Louis, MO, USA) with an essential role in the performance of the visual system. In the stabilization process, Tween 80 (polyethylene glycol monooleate (20), Sorbitan) and CTAB (cetyl-trimethyl-ammonium bromide) have been used as surfactants in order to decrease the superficial tension between the aqueous and the oily phases and increase the stability of the particulate capsules. If Tween 80 is a water-soluble non-ionic hydrophilic surfactant [46], CTAB is borderline between hydrophilic and hydrophobic dispersible agent [47]. The Tween80 and CTAB used for experimental studies were purchased from *Sigma Aldrich*^®^, respectively *Fluka*^®^ (Buchs, Switzerland).

The chitosan (CH) used for experimental studies was low molecular weight type, 80 kDa, with a degree of deacetylation of 75–85%, and purchased from *Sigma Aldrich*^®^. The sodium hyaluronate (HA) used for experimental studies of this work, also low molecular weight type, 40 kDa, and heparin sodium (≥140 USP units/mg) were purchased from *Sigma Aldrich*^®^.

**Figure 13:**
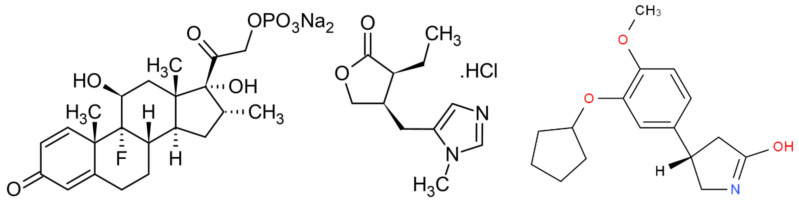
Structure of dexamethasone phosphate (**left**), pilocarpine hydrochloride (**middle**) and bevacizumab (**right**).

The actives incorporated in the core emulsion were dexamethasone phosphate and pilocarpine hydrochloride (Figure 13), and were purchased from *Sigma Aldrich*^®^. Dexamethasone contains dexamethasone phosphate as active substance and is part of a group of pharmaceutical forms called corticosteroids [48]. In ophthalmology, dexamethasone is widely used in the local treatment of various inflammatory conditions: uveitis, keratitis (except epithelial), conjunctivitis, scleritis and episcleritis. In intravitreal administration, dexamethasone is involved in the treatment of macular edema secondary to vascular occlusions, diabetes or uveitis [49]. The half-life of dexamethasone in intravitreal administration is 190 min, and its bioavailability is 80–90% [50]. Pilocarpine is a natural alkaloid extracted from plants of *Pilocarpus species* with activity among cholinergic receptors. The mechanism of action of pilocarpine, through topical application to the eyeball, stimulates contraction of the pupils and ciliary muscle causing a transient decrease in intraocular pressure [51]. Bevacizumab is a monoclonal antibody that functions as an angiogenesis inhibitor.

### 4.2. Preparation of Polymeric Particles

The polymeric particles were prepared by a pre-emulsion coating using the deposition in layers of biopolymers (layer-by-layer) [52,53,54]. Oil in water pre-emulsion was obtained by using an oily solution of retinol acetate (vitamin A) or α-Tocopherol acetate (vitamin E) and a mixture of Tween 80 and CTAB surfactants. The surfactants were used in a mass ratio of 98% and 2% mol/mol, with a weight ratio surfactant/oil solution of 1:1. The aqueous phase was represented by a 5 mM concentration buffer-phosphate solution (PBS, based on NaH_2_PO_4_ × H_2_O and Na_2_HPO_4_ × H_2_O; pH 7.4). Over the oiled mixture an 8-fold volume of PBS was added through an infusion system (infusion rate was 3 drops/10 s) under mechanical stirring, at a speed of 800 rpm.

The layer-by-layer (Lbl) addition technique of polyelectrolytes (chitosan, sodium hyaluronate and heparin sodium) is one of the most effective methods of coating particulated structures. The method consists in the use of polymer solutions with opposite electrical charges, deposited at the same time on different structures. In this paper, an important reference point in the application of the LbL method was the deposition of polymer molecules on cores represented by colloidal particle aggregates. In practice, aqueous solutions of biopolymers of low concentrations were used. Chitosan solution 0.1% (wt/vol) was obtained by dissolution of powder in diluted acetic acid solution (1% wt/vol, in distilled water). Heparine solutions, respectively, sodium hyaluronate, were obtained by dissolution in distilled water (0.5%, wt/vol) and were used in quantities corresponding to a mass ratio of 1:1, oily phase: polymer, and 1:6 for oily phase: chitosan.

For the addition of polymer solutions, the cationic emulsion was diluted 25 times with distilled water, dropwise, under magnetic stirring (800 rpm). First, the solution of polyanonians (sodium hyaluronate or heparin) was added, the system was left under shaking for another 30 min, and then coated by adding the solution of chitosan.

The loading of the particles with drug was performed after the polymer coating step by the layer-by-layer method. The pilocarpine hydrochloride solution (30%, wt/vol) was added dropwise to avoid precipitation in the previously prepared particulate system. The mixture was subjected to magnetic stirring for 30 min at a constant speed of 300 rpm. The dexamethasone phosphate/bevacizumab solutions were loaded using the same procedure.

### 4.3. Characterization of Emulsions and Particles

#### 4.3.1. Particles Size and Zeta Potential

The particles size was measured using the dynamic light scattering (DLS) technique. Samples subjected to DLS analysis were taken at each stage: primary emulsion containing vitamin A or vitamin E, particulate systems coated with hyaluronic acid/chitosan and particles loaded with dexamethasone/pilocarpine. The average particle sizes obtained in the synthesis phases, their distribution and the zeta potential were determined using the *Malvern Zetasizer NanoS*, from Malvern Panalytical Ltd. (Malvern, UK). The dimension measurement analyzer uses the DLS (*dynamic light scattering)* principle, measuring the diffusion of Brownian particles (particles or polymer molecules) and converts it into size and dimension distribution using the Stokes–Einstein relationship. The speed with which the particles of the disperse phase are moving is all the greater the smaller their diameter [55]. On the basis of the displacement speed, the transactional diffusion coefficient is established, and with the help of the Stokes–Einstein equation the hydrodynamic diameter of the particles was determined:

(3) $$D_{H}=\frac{k_{B}T}{3\pi \eta D}$$

where $D_{H}$—hydrodynamic diameter; *D*—diffusion coefficient; $k_{B}$—Botzmann’s constant; *T*—absolute temperature; *η*—environment viscosity.

The zeta potential of the internal phase cells of emulsions as well as the particulate capsules was measured using the *Zetasizer Malvern NanoS* disposition. Thus, by determining electrophoretic mobility, and, on the basis of the Smoluchowski equations, the value of the zeta potential was established [56].

(4) $$\zeta =\frac{\eta \mu }{\varepsilon }$$

where *η*—environment viscosity of dispersion; *μ*—electrophoretic mobility; *ε*—dielectric constant of the dispersion environment.

Due to the electrostatic attraction between the loaded surface of the particles and the ions in the environment, when the particles move into the dispersing medium, they are also moved by a layer of hydrated ions. Between the surface layer of the particles and the ions in the environment, the distribution of the ions takes place on the surface of an electrically charged particle dispersed in a medium with a high dielectric constant in which there are electrolytes of both signs.

#### 4.3.2. Optical Properties of Particles Suspensions

The refraction index of the emulsions and corresponding particles suspensions was measured for sample at dilutions from 0% to 100%. Measuring and obtaining an appropriate refractive index are essential to obtain formulations that do not raise problems of accommodating and blurred vision after instillation of the preparation in the conjunctival sac. The measurements were performed at room temperature (24–25 °C), using an Abbe refractometer (Bausch and Lomb Optical Company, Rochester, NY, USA) by placing one drop of the formulation on the slide. The experiments were performed in triplicate.

#### 4.3.3. Stability of the Emulsions and Particles

The study followed the evolution of the size and zeta potential in time for both emulsion and polymer samples added over long intervals (8–16 months). This study is particularly important to anticipate the time for which the systems will remain stable and the necessary conditions for conditioning and storage. Zeta size and potential changes were determined using the Malvern Zetasizer NanoS device, from Malvern Panalytical Ltd., Malvern, UK.

### 4.4. FT-IR Data

FT-IR spectra were recorded using a Nexus FT-IR Diamond HTR instrument (Thermo Scientific, Waltham, MA, USA) in the range of 400–4000 cm^−1^ using a Smart Orbit ATR accessory with diamond crystal and Omnic 8.0 software, Waltham, MA, USA). The samples were measured in the solid form, in KBr discs (2 % dried particles, *w*/*w*).

### 4.5. In Vitro Drug Release Studies

In vitro release profiles of pilocarpine/dexamethasone from particles were studied in phosphate buffered (PBS) solution (pH 7.4). A volume of 5 mL of emulsion was transferred into a dialysis bag (molecular weight cut-off 12,400 Da) that have been placed in preheated PBS (total volume: 50 mL). The release study was performed in an incubator shaker, at 37 °C for 72 h. At selected time intervals, samples were extracted from the solution outside of the dialysis bag (*n* = 3) for UV–Vis analysis. The drug concentration was measured using a UV1700 PharmaSpec spectrophotometer (Shimadzu, Japan) at the wavelength 241 nm for dexamethasone and 282 nm for pilocarpine. The results were processed using calibration curves. The cumulative released drug concentration was plotted against time. The experiments were repeated three times and the results are expressed as a mean ± standard deviation (SD).

### 4.6. Mathematical Modelling for Drug Release

Conventional, regularly used theoretical models are based on the premise that variables defending the interactions in any generally named polymer–drug complex system are differentiable [57,58] (for details, see the models of Korsmeyer–Peppas, Higuchi, Hixson–Crowell or Weibull [59]). This assumption can be considered rather unjustified as the reality of the interaction is often more complex and defined by borderline nonlinear dynamics. Therefore, the successful implementation of the differentiable models need to be accepted as partially on domains which are defining a clear limit, where the validity of differentiability and integrability is respected. Classical mathematical procedures (differentiable and integrable) are often seen as deficient when relating to the polymer–drug complex system dynamics which are known to presented clear both non-linearity and chaoticity. To incorporate these types of dynamics while simultaneously attempting to use differential mathematical procedures, it becomes mandatory to explicitly add scale resolution into the physical variable definitions and implicitly in the fundamental equations used to express the overall governing dynamics. This paradigm change can be interpreted by adding a dependence on the scale resolution for all variable dependent on space and time coordinates. The approach will also aid the transition from a classical approach to a new mathematical vision with non-differentiability and non-integrability as main characteristics of the systems. On a larger scale it implies the change from operating with a variable defined by non-differentiable function to approximations of the respective functions generated by their averaging at selected scale resolutions. Therefore, all variables assigned to define the polymer–drug complex system dynamics will act as the limit for family of mathematical functions, with the property of being non-differentiable for null scale resolution and differentiable for non-zero scale resolutions [60]. The same approach can be extended within reasonable implementation to similar complex processes involving discharge plasmas [61] or laser produced plasma [62,63]. The similarities are seen in the wide range of entities with different properties which are defined by various spatial and temporal scales.

Developing new methods of characterizing polymer–drug complex system dynamics would require developments towards new geometrical structures. In these models the motion laws which are invariant to space-time transformations, are joined with scale laws which are invariant to space-times scale transformations. The development of such geometrical structure can be founded on concepts such as “multifractality”, and appropriate theoretical models founded on the fractal theory of motion (in the arbitrary and constant fractal dimension). Relevant results regarding the implementation of such model in the complex systems dynamics topic can be seen in [64,65,66,67,68,69,70,71,72].

The core hypothesis of the proposed model considers the structural units of any polymer–drug complex system dynamics as being defined by continuous, but multifractal motion curves (non-differentiable curves). These geometrical objects (multifractal curves) display the self-similarity property in all the containing points which implies the existence of holography (every part reflects the whole) as a main feature of the multifractal systems. Essentially, we are presenting a holographic implementation of polymer–drug complex system structural units dynamics via multifractal “regimes” of Riccati type equations. This approach entails defining the polymer–drug complex system structural units dynamics by using Riccati type equations at various scale resolutions.

The fractal theory of motion from the scale relativity theory developed for the release mechanisms dynamics becomes operational over the scale covariant derivative [72,73]:

(5) $$\frac{\hat{d}}{dt}=\partial_{t}+{\hat{V}}^{l}\partial_{l}+\frac{1}{4}{\left(dt\right)}^{\left(\frac{2}{D_{f}}\right)-1}D^{lp}\partial_{l}\partial_{p},$$

where

$${\hat{V}}^{l}=V_{D}^{l}-V_{F}^{l}$$

$$D^{lp}=d^{lp}-i{\hat{d}}^{lp}$$

(6) $$d^{lp}=\lambda_{+}^{l}\lambda_{+}^{p}-\lambda_{-}^{l}\lambda_{-}^{p}$$

$${\hat{d}}^{lp}=\lambda_{+}^{l}\lambda_{+}^{p}+\lambda_{-}^{l}\lambda_{-}^{p}$$

$$\partial_{t}=\frac{\partial }{\partial t},\;\partial_{l}=\frac{\partial }{\partial x^{l}},\;\partial_{l}\partial_{p}=\frac{\partial }{\partial x^{l}}\frac{\partial }{\partial x^{p}},\;i=\sqrt{-1},\;l,p=1,2,3$$

In Equations (5) and (6), $x^{l}$ represents the fractal spatial coordinate, t is the non-fractal time with the role of motion curves affine parameter, ${\hat{V}}^{l}$ is the complex velocity, $V_{D}^{l}$ is the scale resolution independent differential velocity dt, $V_{F}^{l}$ is the scale resolution dependent non-differentiable velocity, $D_{F}$ is the fractal dimension characterizing the movement curve, $D^{lp}$ is the differentiable–non-differentiable transition associated constant tensor, $\lambda_{+}^{l}\left(\lambda_{+}^{p}\right)$ is the constant vector defining the backward differentiable–non-differentiable processes and $\lambda_{-}^{l}\left(\lambda_{-}^{p}\right)$ is the constant vector associated to forward differentiable–non-differentiable processes. For the fractal dimension several definitions can be used: in the sense of Kolmogorov, in the sense of Hausdorff–Besikovitch, etc. [68,69]. To further the development of our model only one definition must be selected and used for the polymer–drug complex system dynamics. The absolute limitation of this choice is imposed by the constant characteristic of the definition throughout the entirety of the dynamic analysis: i.e., it is generally used for correlative processes $D_{f}<2$, while for non-correlative processes $D_{f}>2$, etc. [68,69].

In the following we will accept the functionality property of the scale covariance principle (i.e., applying the operator Equation (3) to the released drug mass *M*, by not considering any contains from possible external factors) the dynamics can be described though the differentiable equation:

(7) $$\frac{\hat{d}M}{dt}=\partial_{t}M+{\hat{V}}^{l}\partial_{l}M+\frac{1}{4}{\left(dt\right)}^{\left(\frac{2}{D_{f}}\right)-1}D^{lp}\partial_{l}\partial_{p}M=0$$

This means that the temporal variation of the released mass ($\partial_{t}M$), the “fractal convection” of the released drug mas (${\hat{V}}^{l}\partial_{l}M$) and the “fractal dissipation” of the released drug mass ($\frac{1}{4}{\left(dt\right)}^{\left(\frac{2}{D_{f}}\right)-1}D^{lp}\partial_{l}\partial_{p}M$) make their balance in any point of the fractal curve. In particular, if the dynamics is driven through Markov type stochastic processes [68,69] then:

(8) $$\lambda_{+}^{l}\lambda_{+}^{p}=\lambda_{-}^{l}\lambda_{-}^{p}=2\lambda \delta^{lp}$$

where λ is a coefficient associated to the differentiable–non-differentiable transition and $\delta^{lp}$ is the Kronecker’s pseudo tensor:

(9) $$\delta^{lp}=\left\{\begin{array}{c} \;\;1\;\;\;i=l\\ \;0\;\;\;\;i\neq l \end{array}\right.$$

Under these conditions, the differential Equation (8) takes the simple form:

(10) $$\frac{\hat{d}M}{dt}=\partial_{t}M+{\hat{V}}^{l}\partial_{l}M-i\lambda {\left(dt\right)}^{\left(\frac{2}{D_{f}}\right)-1}\partial^{l}\partial_{l}M=0$$

or by separating the drug release dynamics on scale resolutions:

(11) $$\partial_{t}M+{\hat{V}}^{l}\partial_{l}M=0$$

at differentiable scale resolution and

(12) $$-V_{F}^{l}\partial_{l}M-\lambda {\left(dt\right)}^{\left(\frac{2}{D_{f}}\right)-1}\partial^{l}\partial_{l}M=0$$

at non-differentiable scale resolution. Since the release dynamics implies complex dynamics (self-structuring, fickian and non-fickian type diffusion, etc.) at a differentiable–non-differentiable scale (i.e., at a mesoscopic scale), from Equations (6) and (7) by adding them we obtain the fractal diffusion equations:

(13) $$\partial_{t}M+\left(V_{D}^{l}-V_{F}^{l}\right)\partial_{l}M=\lambda {\left(dt\right)}^{\left(\frac{2}{D_{f}}\right)-1}\partial^{l}\partial_{l}M$$

From here, if we use the $V_{D}^{l}\equiv V_{F}^{l}$ condition, which specifies the synchronization of the release kinetics at the two scale resolutions (differentiable and non-differentiable), Equation (13) can be simplified as follows:

(14) $$\partial_{t}M=\lambda {\left(dt\right)}^{\left(\frac{2}{D_{f}}\right)-1}\partial^{l}\partial_{l}M$$

In the one-dimensional stationary case Equation (14) becomes:

(15) $$\frac{d^{2}M}{dx^{2}}+k_{0}^{2}M=0$$

with

(16) $$k_{0}^{2}=\frac{\lambda }{2m_{0}\lambda^{2}{\left(dt\right)}^{\left(\frac{4}{D_{f}}\right)-2}}$$

In Equation (16) λ is a variable separation constant and $m_{0}$ is the rest mass of the polymer–drug structure unit. The solution of Equation (10) can be written in the form:

(17) $$M\left(x\right)=he^{i\left(k_{0}\;x+\theta \right)}+\bar{h}e^{-i\left(k_{0}\;x+\theta \right)}$$

where *h* represents the complex amplitude, $\bar{h}$ is the complex conjugate of *h* and *θ* an arbitrary phase. Hence, *h*, $\bar{h}$ and *θ* define each structural unit of a potential polymer–drug system that exhibits the same *k*_0_, as one of its fundamental properties.

Equation (17) has a “hidden” symmetry based on homographic group of fractal type. The ratio of two independent linear solution of Equation (16), *ε*, represents a solution for Schwartz’s differential equation of fractal type (see [73,74] for the classical case)

(18) $$\left\{\epsilon ,x\right\}=\frac{d}{dx}\left(\frac{\ddot{\epsilon }}{\dot{\epsilon }}\right)-\frac{1}{2}{\left(\frac{\ddot{\epsilon }}{\dot{\epsilon }}\right)}^{2}=2k_{0}^{2}$$

(19) $$\dot{\epsilon }=\frac{d\epsilon }{dx},\ddot{\epsilon }=\frac{d^{2}\epsilon }{dx^{2}}$$

The left side of Equation (18) is invariant to the homographic transformations (fractal type):

(20) $$\epsilon ↔\epsilon^{\prime }=\frac{a\epsilon +b}{c\epsilon +d}$$

with *a*, *b*, *c* and *d* real fractal parameters. The relation, Equation (18), defines the fractal SL(2R) group when considering to all possible values of these fractal parameters.

Thus, all the polymer drug structural units defined by identical *k*_0_ are in biunivocal relation with the transformations of the fractal SL(2R) group. This permits the development of a unique parameter of fractal type *ε* for each polymer drug structural unit, separately. Indeed, as a “guide” it is chosen the general form of the solution of Equation (16), which is written as

(21) $$\epsilon^{\prime }=l+m\;\tan\left(k_{0}x+\theta \right)$$

Thus, by tailoring *l*, *m* and *θ* it is conceivable to describe any and all polymer drug structural units. In such an approximation, identifying the phase from Equation (19) with Equation (15), the multifractal key parameter becomes:

(22) $$\epsilon^{\prime }=\frac{h+\bar{h}\epsilon }{1+h},\;h=l+im,\bar{h}=l-im,\;\epsilon \equiv e^{2i\left(k_{0}x+\theta \right)}$$

It can be seen that Equation (21) is a solution of Equation (18) which implies, by explicating Equation (20), the fractal SL(2R) group:

(23) $$h^{\prime }=\frac{ah+b}{ch+d},\;{\bar{h}}^{\prime }=\frac{a\bar{h}+b}{c\bar{h}+d},\;k^{\prime }=\frac{c\bar{h}+b}{ch+d}\;k$$

Therefore, the group (20) works as “synchronization modes” between the different polymer drug structural units. the synchronization implies a connection between the amplitudes and phases of each structural unit. More precisely, through the group (20), the phase of *k* can only be varied with quantities which are dependent on the polymer drug structural units amplitude at the transition among various polymer drug structural units. Moreover, the polymer drug structural unit amplitude can be influenced a homographic perspective. The known “synchronization” which manifests through the delay of the amplitudes and phases are representing here only a very particular case.

The structure of group (20) is typical of SL(2R) one, which we take in the standard form

[A_1_, A_2_] = A_1_, [A_2_, A_3_] = A_3_, [A_3_, A_1_] = −2A_1_ (24)

where A_k_, k = 1, 2, 3 are the infinitesimal generators of the group. Due to the simple transitivity property of the group, the generators can be found as the part of the Cartan multifractal coframe (see [74] for the classical case) from:

(25) $$d\left(f\right)=\sum \frac{\partial f}{\partial x^{k}}dx^{k}=\left\{\begin{array}{c} \omega^{1}\left[h^{2}\frac{\partial }{\partial h}+{\bar{h}}^{2}\frac{\partial }{\partial \bar{h}}+\left(h-\bar{h}\right)k\frac{\partial }{\partial k}\right]+\\ +2\omega^{2}\left(h\frac{\partial }{\partial h}+\bar{h}\frac{\partial }{\partial \bar{h}}\right)+\omega^{2}\left(\frac{\partial }{\partial h}+\frac{\partial }{\partial \bar{h}}\right) \end{array}\right\}$$

where *ω^k^* are the parts of the Cartan fractal coframe computed from the system:

(26) $$dh=\omega^{1}h^{2}+2\omega^{2}h+\omega^{3}\;d\bar{h}=\omega^{1}{\bar{h}}^{2}+2\omega^{2}\bar{h}+\omega^{3}\;dk=\omega^{1}\left(h-\bar{h}\right)$$

Thus, we have can obtain the infinitesimal fractal generators and the fractal coframe by connecting the right-hand side of Equation (25) with the classic dot product of fractal SL(2R) algebra

(27) $$\omega^{1}A_{3}+\omega^{3}A_{1}-2\omega^{2}A_{2}$$

so that

(28) $$A_{1}=\frac{\partial }{\partial h}+\frac{\partial }{\partial \bar{h}},\;A_{2}=h\frac{\partial }{\partial h}+\bar{h}\frac{\partial }{\partial \bar{h}},\;A_{3}=h^{2}\frac{\partial }{\partial h}+{\bar{h}}^{2}\frac{\partial }{\partial \bar{h}}+\left(h-\bar{h}\right)k\frac{\partial }{\partial k}$$

and

(29) $$\omega^{1}=\frac{dk}{\left(h-\bar{h}\right)k},2\omega^{2}=\frac{dh-d\bar{h}}{h-\bar{h}}-\frac{h+\bar{h}}{h-\bar{h}}\frac{dk}{k},\;\omega^{3}=\frac{hdh-hd\bar{h}}{h-\bar{h}}+\frac{h\bar{h}dk}{\left(h-\bar{h}\right)k}$$

We should mention that, in [68,69,70,71,72], it does not work with the previous differential forms, but with the absolute invariant differentials

(30) $$\omega^{1}=\frac{dk}{\left(h-\bar{h}\right)k},\omega^{2}=i\left(\frac{dk}{k}-\frac{dh+d\bar{h}}{h-\bar{h}}\right),\;\omega^{3}=\frac{kd\bar{h}}{h-\bar{h}}$$

The major benefit of using this representation is seen in the connections with the Poincare representation of the Lobachevsky plane. The metric is given by

(31) $$\frac{ds^{2}}{g}={\left(\omega^{2}\right)}^{2}-4\omega^{1}\omega^{2}={\left(\frac{dk}{k}-\frac{dh+d\bar{h}}{h-\bar{h}}\right)}^{2}+4\frac{kd\bar{h}}{h-\bar{h}}$$

where *g* is a constant.

The presented metric can be simplified to that of Poincare for *ω*^2^ = 0. This clearly is defined by *θ* variable as the parallelism angle, understood in Levi-Civita sense, of the hyperbolic fractal plane (see [73,74] for the connection of the fractal type). Let us return to homographic fractal transformation (18). All defined polymer–drug system structural units, when a parallelism direction, in Levi-Civita sense, turns functional on the manifold induced by SL(2R) fractal group, can be defined through 4 homogenous coordinates (*a*, *b*, *c* and *d*) or 3 non-homogenous coordinates. At the same time by imposing the condition that free polymer–drug system structural units can be uniquely characterized from a Riccati fractal equation in multifractal differentials which we will call Riccati gauge of fractal type:

(32) $$d\frac{a\varepsilon +b}{c\varepsilon +d}=0$$

it implies

(33) $$d\varepsilon =\omega^{1}\varepsilon^{2}+\omega^{2}\varepsilon +\omega^{3}$$

where *ω*^1^, *ω*^2^ and *ω*^3^ are the components of the Cartan fractal coframe extracted from relations (29). Hence, in order to describe any polymer–drug system dynamics as a sequence of states containing an ensemble of simultaneous structural units, it only requires to define three differentiable 1-forms, describing a coframe of SL(2R) multifractal algebra. Accordingly, a particular state of a polymer–drug system in a defined dynamics could be systematized as a metric plane space (a Riemannian multifractal three-dimensional space). Furthermore, the geodesics of Riemannian fractal space can be given through some fractal conservations equations:

(34) $$\omega^{1}=a^{1}d\tau ,\;\omega^{2}=a^{2}d\tau ,\;\omega^{3}=a^{3}d\tau$$

where *a*^1^, *a*^2^ and *a*^3^ are constants and *τ* represents the geodesics affine parameter. This means that, along these geodesics defined by differential Equation (30) is an ordinary differential of Riccati type

(35) $$\frac{d\varepsilon }{d\tau }=a^{1}\varepsilon^{2}+2\omega^{2}\varepsilon +a^{3}$$

Let us consider the following form:

(36) $$A\frac{d\varepsilon }{d\tau }-\varepsilon +2B\varepsilon +AC=0$$

where

(37) $$A=\frac{1}{a^{1}},\;B=-2\frac{a^{2}}{a^{1}},\;AC=-\frac{a^{3}}{a^{1}}$$

Since the polynom roots

(38) $$P\left(\varepsilon \right)=\varepsilon^{2}+2B\varepsilon -AC$$

can be given:

(39) $$\varepsilon_{1}=B+iA\cdot \Omega ,\;\varepsilon_{2}=B-iA\cdot \Omega ,\;\Omega^{2}=\frac{C}{A}-{\left(\frac{B}{A}\right)}^{2}$$

the change of variable

(40) $$z=\frac{\varepsilon -\varepsilon_{1}}{\varepsilon -\varepsilon_{2}}$$

transforms Equation (36) in

(41) $$\dot{z}=2i\cdot \Omega z$$

of solution

(42) $$z\left(\tau \right)=z\left(0\right)e^{2i\Omega \tau }$$

As such, if the initial condition *z*(0) is adequately defined, it gives the possibility to develop the general solution of Equation (35), by giving the transformation (37) the form:

(43) $$\varepsilon =\frac{\varepsilon_{1}+re^{2i\Omega \left(\tau -\tau_{0}\right)}}{1+re^{2i\Omega \left(\tau -\tau_{0}\right)}}$$

where *r* and *τ*_0_ are integration constants. By using Equation (40), one can give the solution in real terms:

(44) $$z=B+A\Omega \cdot \left(\frac{2r\;sin\left[2\Omega \left(t-t_{0}\right)\right]}{1+r^{2}+2r\;cos\left[2\Omega \left(t-t_{0}\right)\right]}+i\frac{1-r^{2}}{1+r^{2}+2r\;cos\left[2\Omega \left(t-t_{0}\right)\right]}\right)$$

### 4.7. In Vitro Tests for Ocular Tolerability

In vitro ocular tolerability testing of active substance-loaded polymeric formulations was evaluated using a modified HET-CAM assay (chicken egg chorioallantoic membrane assay). The method is based on the use of incubated chicken eggs. The eggs were lettered on their surface and incubated at 37 °C ± 0.5 °C and 40% ± 5% humidity for 7 days [75]. The eggs were laid and incubated in a horizontal position to ensure the correct positioning of the embryo. During the test, at least 3 times a day, the samples were rotated manually to ensure the correct development and viability of the embryo. On the 9th day of incubation, the eggs were checked by positioning in the light to ensure fertility, and the shell was marked on the airspace line. After the 10 full days of incubation, each egg was individually removed from the oven and placed in a support system being positioned with the larger surface facing up. The shell was sectioned with a scalpel and removed just above the marked line of the chorioallantoic membrane (CAM). After removal of this section, the inner membrane which is in direct contact with CAM was moistened dropwise with 2 mL of 3% saline. The inner membrane was carefully removed using tweezers, without damaging the blood vessels, revealing the chorioallantoic membrane.

A volume of test solution was added directly to the chorioallantoic membrane, and a timer was started. At various times, for a period of 5 min after application of the test nanoemulsion any effect (lysis, hemorrhage or coagulation) was observed and compared with the standard (3% saline (negative) and sodium hydroxide solutions (positive)). Qualitative data were recorded by photographing the samples at different time intervals to quantify vascular damage, allowing for a more detailed and robust analysis of active pharmaceutical systems. At the end of the 5 min period, the membrane is considered to have been disturbed. Each test was performed in triplicate. The HET-CAM test is considered useful as a model for ophthalmic tissue, especially connective tissue, due to the properties of the chorioallantoic membrane (CAM). Being a functional membrane, supplemented with a vascular and inflammatory response, the HET-CAM test assesses the ability of a test substance to damage blood vessels and cause bleeding, coagulation, hyperemia or lysis [76]. Based on reaction time, an irritation score (IS) is calculated (Table 2).

The time (s) of onset was noted for each of the irritating effects (hemorrhage, hyperemia and coagulation) [76] and the irritation potential (IS) was calculated using the following Equation (45) [77,78]:

(45) $$IS=\frac{\left(301-h\right)x5}{300}+\frac{\left(301-l\right)x7}{300}+\frac{\left(301-c\right)x9}{300}$$

where *h*—reaction time (s) for hemorrhage; *l*—reaction time (s) for lysis/hyperemia; *c*—time (s) of reaction for coagulation.

## 5. Conclusions

Double-emulsion method was used to obtain particles based on biopolymer (heparin/hyaluronic acid, chitosan) with hydrophobic core (vitamin A/vitamin E) and incorporation of drugs (dexamethasone phosphate/pilocarpine hydrochloride/bevacizumab). The obtained particles were stable in shape and size (in the range 200–300 nm), due to the covalent and ionic interactions polymer–polymer and polymers–drug. The particles do not aggregate, and as dispersion have a low polydispersity. In vitro tests indicated for the irritation score values that included the prepared particulate systems in the “non-irritating” spectrum.

From the kinetic curves of dexamethasone/pilocarpine/bevacizumab release it was observed that the increase of the concentration of the drug is directly proportional to the increase of the release time. The in vitro kinetic study of the controlled release process of active substances, demonstrates the ability of this type of polymeric system to release in a controlled manner, highlighting the superiority of this regimen of drug administration compared to the conventional methods.

A theoretical model has been developed based on a multifractal paradigm. The model captured various drug release scenario expressed through period doubling, damped oscillations, quasi-periodicity or intermittency. Among these, a special mode of Kink type, better reflects the empirical data. The fractal study indicated more complex interactions between the angiogenesis inhibitor bevacizumab and polymeric structure.

## Figures and Tables

**Table 1:** Size (nm) and zeta potential (mV) for pre-emulsions prepared with different concentrations of CTAB and vitamin A.

Pre-Emulsion	CTAB (%)	Size (nm)	PDI	Zeta Potential (mV)
PE0	0	181 ± 33	0.072 ± 0.015	−6.92 ± 0.8
PE2	2	136 ± 12	0.153 ± 0.040	+36.9 ± 8.4
PE4	4	168 ± 23	0.090 ± 0.021	+12.8 ± 1.5
PE5	6	174 ± 29	0.171 ± 0.027	+49.5 ± 5.2

**Table 2:** Scoring scale for the HET-CAM test.

Effect	Score			Cumulative Score	Irritation Degree
	0.5 min	2 min	5 min	0–0.9	Non-irritant
Lysis/Hyperemia	5	3	1	1.0–4.9	Less irritant
Hemorrhage	7	5	3	5.0–8.9	Moderate irritant
Coagulation	9	7	5	9.0–21.0	Sever irritant

## Data Availability

Not applicable.
